# Adding a ‘+’ to ‘EBM’ Is Insufficient and Misleading

**DOI:** 10.1111/jep.70720

**Published:** 2026-11-04

**Authors:** Trisha Greenhalgh

**Affiliations:** ^1^ Nuffield Department of Primary Care Health Sciences University of Oxford Oxford UK

In 1979, a Canadian Task Force published an evidence review which included a brief account of how the team had prioritized studies on the effectiveness of interventions: they had ranked well‐designed randomized controlled trials (RCTs) highest, then well‐designed cohort or case‐control studies, then other comparative studies, then expert opinion [1]. Never did a well‐argued, fully justified paragraph in an academic paper become more widely misinterpreted, mis‐cited, and overextended. The ‘hierarchy of evidence’, as this list became known, morphed into an excuse for privileging the RCT for any research question; for assuming that an RCT, however bad, should be preferred over an observational study, however good; and for consigning all non‐RCT evidence to the not‐even‐looked‐at pile. One casualty of this shift in what counted as high‐quality evidence was *mechanistic* evidence, including studies of pathophysiology, biochemical and pharmacodynamic interactions, human behaviour, and operational processes. EBM, at its lowest point and in the most naïve hands, became a rigid, rule‐bound, theory‐free, RCT‐only zone.

Against this backdrop, the emergence of the EBM+ Consortium in 2017 was a distinct step in the right direction. This interdisciplinary group included philosophers, basic scientists, epidemiologists and clinicians. Building on an earlier philosophical paper by Russo and Williamson [2] and on foundational work in medical research by Bradford Hill [3], they argued that to demonstrate causality, two kinds of evidence are usually needed: evidence of *association* or *correlation* (e.g., that a change in one variable is associated with another) and evidence of *mechanism* (a physical, behavioural or social means by which this change is achieved) [4]. They also argued that these two kinds of evidence are complementary, not hierarchically related and that both kinds of evidence should be examined fully. It makes no sense to say that RCT evidence of a drug's efficacy is *better* than a study demonstrating *how* the drug works at a molecular level. Both are needed.

While some detractors cited cautionary tales of plausible‐sounding mechanisms that ultimately proved misleading [5, 6, 7], and others insisted that the ‘+’ was a retrograde step that would compromise the ‘rigour’ of EBM [8], the arguments of the EBM+ Consortium were, by and large, persuasive. It is hard to dispute, for example, that in the drug approval pathway, evidence of efficacy from RCTs should be complemented by mechanistic studies in “drug discovery and development; assessing pharmaceutical quality; devising dosage regimens; assessing efficacy, harms, external validity, and cost‐effectiveness; evaluating adherence; and extending product licences” (page 1166) [9].

The plus sign in EBM+ does rhetorical work. It implies, firstly, that mechanistic evidence takes nothing away from traditional EBM (on the contrary, the syntax implies, it is entirely consistent with the EBM tradition and *adds* to it), and secondly, the associative evidence generated from (typically) RCTs sits *separately* from mechanistic evidence. The initial methodology described for EBM+ was to (a) identify and analyse associative evidence, (b) identify and analyse mechanistic evidence, and then (c) bring these streams of evidence together.

Illari and Russo's paper in this issue uses the example of vitamin D deficiency to show that while the underlying premise of EBM+ (i.e., that both associative and mechanistic evidence are needed for demonstrating causality) is sound, the specific methodology of aligning them (analyse separately, then combine) is not [10]. Their argument can be summarized as follows. Any correlational study, including the best‐conducted RCT, requires someone to decide *which variables to study* and *how and when to measure them*. Those decisions rest on what is currently believed about mechanisms. Evidence of mechanism is therefore necessary not merely *alongside* evidence of correlation but *as an integral part of the analysis* of such correlation. Without evidence of mechanism, you cannot know the worth of the correlation (or lack of correlation) you observe.

At first glance, the vitamin D case looks trivial. Serum is sent to a laboratory and a number comes back. Illari and Russo show that four separate decisions are needed to generate that number: [1] which metabolite to target (25(OH)D, since the active 1,25(OH)2D is unstable and rises misleadingly as vitamin D becomes scarce); [2] what serum concentration constitutes deficiency, which remains contested; [3] whether the bioavailable fraction matters, given that vitamin D binding protein levels themselves vary; and [4] which assay to use, among immunoassay, radioimmunoassay, tandem mass spectrometry and C3‐epimer measurement. Each decision has co‐evolved with changing mechanistic knowledge. Historically, when only the skeletal effects of vitamin D deficiency were under consideration, the differences between assays were immaterial. Once vitamin D is implicated in cardiovascular disease, cancer, infection and liver disease, they may not be.

Two aspects of this case are noteworthy. First, the topic is messy and contested rather than clear‐cut, which allows the authors to show that partial and unsettled mechanistic knowledge still does evidential work. Second, Illari and Russo observe that the most valuable output of their Clinical Research Table may be to reveal what is *not* known.

Some readers will interpret Illari and Russo's paper as a straightforward refinement of EBM+ , adding a caveat about measurement to a framework that is otherwise intact. That reading understates the paper's substantive philosophical contribution. Any procedure that requires evidence of correlation and evidence of mechanism to be appraised separately and then combined presupposes that they are separable. Illari and Russo show that they are not, and that to talk of ‘correlational studies’ and ‘mechanistic studies’ is artificial. All so‐called associative (correlational) studies contain within them multiple mechanistic assumptions. In their words, associative and mechanistic evidence are *intertwined*.

An important consequence of this intertwining concerns sequence. If mechanistic assumptions are built into the design of a study that will subsequently be classified as correlational, then appraising the correlation first and turning to mechanism afterwards leaves key elements of the study unexamined.

A classic example is the MRC Vitamin Study of periconceptional folic acid, in which supplementation began before conception and continued only to the 12th week [11]. This window was selected because the neural tube closes by around the 28th day of gestation, frequently before a woman knows she is pregnant. A trial that was identical in design but which provided supplementary folic acid from the first antenatal visit would have produced a null result however many women it enrolled because it would have *bypassed the causal mechanism* and hence been incapable of examining the causality question. The difference between the two designs rests on a time‐dependent embryological mechanism. Appraising such a study without taking account of mechanistic evidence would have generated a false conclusion of inefficacy.

Table 1 shows this classic folate RCT along with six other examples where mechanistic concerns were crucial in determining what a RCT was capable of showing. In each case, the design decision is invisible to an appraisal that begins with the association. These mechanisms operate at different levels: when the intervention is given, how the population or exposure is defined, and how and when the outcome is measured.

**Table 1:** Mechanistic decisions embedded in the design of correlational studies.

Case	Key mechanistic knowledge influencing RCT design	Critical design decision	How assessing association without mechanism would mislead
Antenatal corticosteroids to prevent hyaline membrane disease of newborn (1972) [12]	Surfactant induction takes about 24 h and wanes after about a week	The key exposure variable is the treatment‐to‐delivery interval	If exposure is coded as ‘ever received’, the estimate will be biased toward null, but the risk‐of‐bias assessment will not register this
Periconceptional folic acid to prevent neural tube defects (1991) [11]	The neural tube closes by around day 28, often before pregnancy is recognised	When the intervention is given	If the window for embryonic development is not covered by the intervention, a null result is mechanistically inevitable
Adjuvant tamoxifen in breast cancer (meta‐analysis published 2011) [13]	Tamoxifen acts on the oestrogen receptor, which receptor‐poor tumours do not express	Who counts as eligible for the drug	If receptor status is not measured or taken account of, the effect is a diluted average across two populations. Efficacy will be underestimated in receptor‐rich patients and over‐estimated in receptor‐poor ones
HbA1c as trial outcome (review published 2014) [14]	Glycation accumulates over the lifespan of the red blood cell, which varies with iron deficiency, haemoglobinopathy, renal disease and pregnancy	Whether to rely on HbA1c as a measure of diabetes control	The same numerical value of HbA1c carries different meanings in different populations. In some, numerical values are unreliable.
Checkpoint inhibitors: pseudoprogression and iRECIST (guideline published 2017) [15]	Immune infiltration can transiently enlarge cancerous lesions, and responses are delayed	What counts as the event (‘progression’), and when it is assessed	RECIST‐defined progression may misclassify responders as failures. Delayed separation of survival curves breaks proportional hazards, so the hazard ratio is itself the wrong summary.
PD‐L1 as an eligibility variable (study 2017 [16]; meta‐analysis 2023 [17])	Expression of PD‐L1 is dynamic and spatially heterogeneous. Antibody clones, platforms and scoring conventions are not interchangeable	Which analyte, which assay, which threshold	‘PD‐L1 positive’ is not one variable; studies using different assays are not comparing like with like
Smoking cessation: the Russell Standard—self‐reported continuous abstinence at 6 m confirmed by a biochemical test (2005) [18]	Relapse is concentrated in the weeks immediately after quitting; self‐reported quitting is unreliable; cotinine, the usual confirmatory biomarker, is a metabolite of nicotine	How abstinence is defined, when it is assessed, and which biomarker confirms it	4‐week self‐reported quit rate and 6‐month verified abstinence are different outcomes (but risk‐of‐bias assessment will not detect the difference). Trials of nicotine replacement must switch to expired carbon monoxide, because NRT will produce cotinine test‐positives

In the smoking cessation example, the difficulty is recursive. The field settled on biochemically verified continuous abstinence at 6 months because of what was known about the natural history of dependence. It then had to settle on which biochemical marker, because the obvious one (cotinine) is generated by the treatment itself. Both decisions are mechanistic and both precede any measurement of association.

If mechanism determines what a study is able to measure, it bears on studies that found nothing as much as on studies that found something. In Illari and Russo's own case of vitamin D deficiency, consistent evidence from randomised trials for benefits of general vitamin D supplementation has not materialised. Bouillon and colleagues, whom they cite, observe that the large recent trials recruited participants who were mostly vitamin D replete at baseline [19], and suggest that this may account for the null results. Whether replete recruitment is a fatal flaw or a reasonable pragmatic choice turns on a mechanistic question: whether the postulated effect is one of repletion, operating only below some threshold, or a graded effect across the range. That is one of the four mechanism‐informed decisions the paper examines. Appraise the association first and you record several large, well‐conducted, null trials. It is only when the mechanism is examined that you learn what those trials were in a position to detect.

An important strength of the tools offered by Illari and Russo is that they serve as an orienting framework but they do not purport to replace human judgement. Nothing is scored; the red flags exist to draw a panel's attention, and it is for domain experts to decide whether a flagged feature is a defect or a sensible choice given the state of the science. This approach treats expertise as an essential component of appraisal rather than as a downstream check on it. It is also a concession that no procedure will deliver the judgement for you.

While the Illari‐Russo paper is persuasive on the intertwining of associations and mechanisms, it leaves room for further extensions. One potential extension is appraisal of mechanistic evidence itself. EBM constructed an elaborate apparatus for judging evidence of association: risk‐of‐bias tools, GRADE, reporting standards. Nothing comparable exists for mechanism. The tables presented in Illari and Russo's paper record whether a mechanism is posited, whether the cited literature supports it, and whether measures are comparable across studies. They do not adjudicate between mechanistic accounts of similar surface plausibility, and Howick's warning about just‐so stories [6] remains largely unanswered. A framework that requires mechanistic evidence to be brought in, without supplying a way of weighing what is brought, risks lending the authority of a method to whichever account is nearest to hand.

Another potential extension is the treatment of bias and confounding. Illari and Russo apply mechanistic scrutiny to the pathway of interest. But non‐adherence, crossover, exposure misclassification and measurement error are themselves causal processes, with direction and magnitude, and they too determine what a study could have detected. If mechanistic appraisal is owed to the mechanism through which an intervention is supposed to act, it is equally owed to the mechanisms that shape what the study could see. I address the treatment of mechanisms of bias in a separate paper [20].

In conclusion, the instinct behind the EBM+ movement was correct. But Illari and Russo have shown that the ‘+’ in ‘EBM+’ is not only insufficient but misleading, since the correction is not additive but constitutive. It requires asking of every correlational study *what mechanistic assumptions are embedded in its variables, its timing and its biases*, and treating the answer as part of the appraisal of the correlation rather than as a separate exhibit. That is a more demanding programme than adding a plus sign to an acronym, and a substantially more promising one.

## Conflicts of Interest

TG is a co‐investigator with Jon Williamson and Rebecca Helm on the UKRI *Interdisciplinary Systematic Review: Mechanistic Evidence and Epistemic Justice* project. She is also a member of Independent Sage.

## Data Availability

No empirical data were generated or used in this paper, which is a commentary on a paper in this issue.
